# Sleep Endoscopy with Positive Airway Pressure: A Method for Better Compliance and Individualized Treatment of Patients with Obstructive Sleep Apnea

**DOI:** 10.3390/life12122108

**Published:** 2022-12-15

**Authors:** Michaela Masárová, Petr Matoušek, Ondřej Jor, Vilém Novák, Adéla Vrtková, Vojtěch Kubec, Karol Zeleník, Pavel Komínek, Martin Formánek

**Affiliations:** 1 Department of Otorhinolaryngology and Head and Neck Surgery, University Hospital Ostrava, 708 52 Ostrava, Czech Republic; 2 Faculty of Medicine, University of Ostrava, 703 00 Ostrava, Czech Republic; 3 Department of Anesthesiology, Resuscitation and Intensive Medicine, University Hospital Ostrava, 708 52 Ostrava, Czech Republic; 4 Department of Pediatric Neurology, University Hospital Ostrava, 708 52 Ostrava, Czech Republic; 5 Department of Applied Mathematics, Faculty of Electrical Engineering and Computer Science, VSB-Technical University of Ostrava, 708 00 Ostrava, Czech Republic; 6 Department of Otorhinolaryngology, Faculty of Medicine in Pilsen, University Hospital in Pilsen, Charles University, 305 99 Pilsen, Czech Republic

**Keywords:** obstructive sleep apnea, drug-induced sleep endoscopy, positive airway pressure

## Abstract

In this study, we aimed to observe the effects of positive airway pressure (PAP) on individual levels of obstruction during drug-induced sleep endoscopy (DISE) of the upper airways (UA), to evaluate at which pressures the obstruction disappeared or worsened, and to identify cases in which PAP was ineffective. This prospective study was conducted from June 2018 to June 2022. PAP testing was performed during DISE in patients with moderate and severe OSA. The pressure was gradually increased over the range from 6.0 to 18.0 hPa. Our findings were evaluated using the VOTE classification. The examination was performed in 56 patients, with a median apnea–hypopnea index (AHI) of 26.4. Complete obstruction of the soft palate was observed in 51/56 patients (91%), oropharyngeal obstruction in 15/56 patients (27%), tongue base obstruction in 23/56 patients (41%), and epiglottic collapse in 16/56 patients (29%). PAP was most effective in cases of complete oropharyngeal obstruction, and least effective in cases of epiglottic collapse, where it was ineffective in 11/16 patients. DISE with PAP is a simple diagnostic method that can be helpful for identifying anatomic and dynamic reasons for PAP intolerance. The main indication is ineffective PAP treatment.

## 1. Introduction

For adults with moderate and severe obstructive sleep apnea (OSA), treatment with positive airway pressure (PAP) is currently considered the “gold standard” [1,2]. Despite this consensus, some of the patients show poor compliance for a variety of reasons—including psychological reasons, allergic contact dermatitis to the mask material, latex allergy, anatomical abnormalities, rhinitis, etc. Additionally, PAP is ineffective in some patients, or works only at intolerably high pressures [3,4]. These patients are then exposed to the risks associated with untreated OSA, which can be life-threatening. In addition, it is assumed that the application of PAP affects all areas of the upper respiratory tract equally, regardless of the individual characteristics of the patient. Therefore, factors such as the location and range of obstruction may often be ignored [4,5].

Drug-induced sleep endoscopy (DISE) is currently the main diagnostic method for identifying sites of obstruction in patients with OSA [6,7]. Since it is mainly performed before planned surgical treatment [7,8], the majority of PAP-treated OSA patients do not undergo DISE [4,5].

PAP performed during DISE is a new combination of diagnostic methods that allows the direct visualization and evaluation of how PAP affects individual collapsing areas of the airway. This method can provide a better understanding how PAP actually works, and possibly predict a good response to and compliance with future PAP treatment [4,5,9,10,11].

A limited number of studies have dealt with this issue. According to the preliminary results of these studies, it appears that overpressure ventilation is more effective in cases of obstruction of the soft palate and oropharynx compared to obstructions of the base of the tongue or cases of epiglottic collapse. These results logically suggest that for certain sites of obstruction, surgical treatment might be more appropriate compared to PAP, regardless of factors such as AHI, BMI, etc. [5,10,11].

In the present study, we aimed to observe the effects of PAP on individual levels of airways obstruction during DISE, to evaluate the reaction of obstruction upon overpressure, and to identify cases in which PAP is ineffective.

## 2. Materials and Methods

This prospective study was performed in accordance with the Declaration of Helsinki and the requirements of Good Clinical Practice, and was approved by the Ethics Committee of the University Hospital Ostrava (identifier: 360/2021). The study was registered in ClinicalTrials under the number NCT02855515. Written informed consent was obtained from each patient before any procedure was initiated.

### 2.1. Study Design and Patients

This prospective study was performed at the tertiary referral center University Hospital Ostrava from June 2018 to June 2022. We consecutively enrolled adult patients with moderate or severe OSA with an apnea–hypopnea index (AHI) of ≥15 episodes/hour. Patients were excluded if they had major comorbidities representing excessive risk for general anesthesia (decompensation phase)—cardiac, liver, or kidney disease, cancer; craniofacial malformations; neurological pathologies; if they were pregnant; or if they did not agree to be included in the study.

Sixty-four consecutive patients were included in the study; eight patients were excluded and examination was performed in fifty-six patients.

### 2.2. Clinical Evaluation

Patients were evaluated by collection of a comprehensive history that covered sleep habits and disturbances. As a subjective measure of a patient’s sleepiness, the Epworth sleepiness scale (ESS) was used. Clinical evaluation included a complete head and neck examination. The upper airways and digestive tract were examined using a flexible videoendoscope with a 3.5 mm diameter (Olympus, Tokyo, Japan).

### 2.3. Drug-Induced Sleep Endoscopy

Sleep endoscopy was performed in the operating room. Intramuscular administration of Dormicum (midazolam) 5 mg and atropine 0.5 mg was performed 30 min before the sleep endoscopy. Subsequently, the patient was induced to sleep with intravenous propofol (an initial 1 mg/kg bolus, followed by 20–30 mg every 3–5 min). The depth of anesthesia was measured using the bispectral index. Vital signs were monitored. Sleep endoscopy was performed using a flexible videoendoscope with a diameter of 3.5 mm (Olympus, Tokyo, Japan). The examination length was 15–20 min. The results were evaluated using the Kezirian VOTE classification, for which obstruction is evaluated in the four localities of the upper airways [12].

### 2.4. Positive Airway Pressure Titration during the Sleep Endoscopy

**Figure 1:**
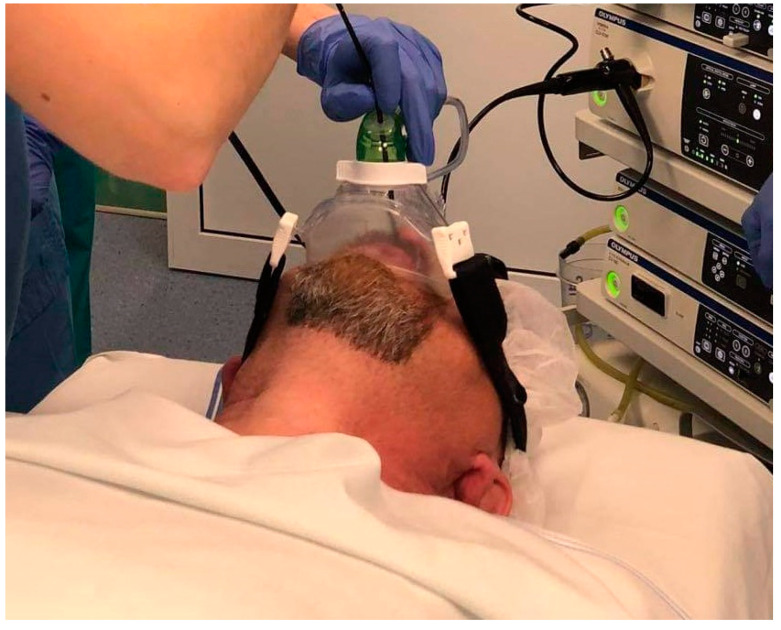
Drug-induced sleep endoscopy (DISE) with simultaneous positive airway pressure (PAP); external view.

Titration was performed using the BiPAP A40 (Philips Respironics, Florida, USA) in PAP mode. PAP titration was performed immediately after DISE. An appropriately sized overpressure ventilation mask (Respironics PerforMax Full-face mask; Philips Respironics, Florida, USA) was applied to the patient’s face. A special connecting valve (Philips Respironics, Florida, USA) was inserted between the mask and the device hose, through which a flexible endoscope was inserted into the nasopharynx and upper airways (Figure 1).

**Figure 2:**
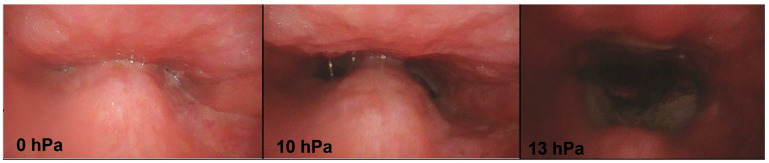
The effect of positive airway pressure (PAP) during drug-induced sleep endoscopy (DISE) at different pressures; endoscopic view.

Sleep endoscopy was then performed under overpressure ventilation. The examination started at a pressure of 6.0 hPa. The pressure on the PAP was gradually elevated (in the range of 6.0, 8.0, 10.0, 12.0, 14.0, and 18.0 hPa (Figure 2). The efficiency of treatment was visually assessed, with simultaneous monitoring of the blood oxygen saturation. At each tested PAP pressure, the VOTE classification was used for evaluation [12].

We compared the findings among the examined patients. Notably, we observed which areas of the upper airways responded better to PAP treatment and in which areas PAP had worse effects.

### 2.5. Statistical Analysis

Numerical variables were presented as the median and interquartile range (IQR). Categorical variables were presented as absolute and relative frequencies (%). The chi-square test for equality of proportions was used for the comparison of the effect of PAP. The significance level was set to 0.05 and all statistical analyses were performed using R software (version 4.2.1).

## 3. Results

### 3.1. Patients Characteristics

This study included a total of 56 patients (9 women, 47 men) aged 22–59 years. The median AHI was 26.4, and median BMI was 29.2 kg/m^2^ (Table 1). During DISE, complete obstruction was observed in the soft palate region in 51/56 patients (91%), in the oropharynx in 15/56 patients (27%), in the tongue base in 23/56 patients (41%), and due to the epiglottis (epiglottic collapse) in 16/56 patients (29%) (Table 2).

### 3.2. Effect of PAP

PAP was most effective in complete oropharyngeal obstruction, where we observed improvement in all 15 (100%) obstructions. PAP was least effective in epiglottic collapse, with improvement observed in only 5/16 cases with obstruction due to the epiglottis. PAP was ineffective in the remaining 11/16 cases with epiglottic collapse (Table 2).

Single-level obstruction was detected in 3/56 patients (5%), and multilevel obstruction (two or more regions) in 53/56 patients (95%). Obstruction was observed at three sites in 25/56 patients (45%), and at all four locations in 11/56 patients (20%).

**Figure 3:**
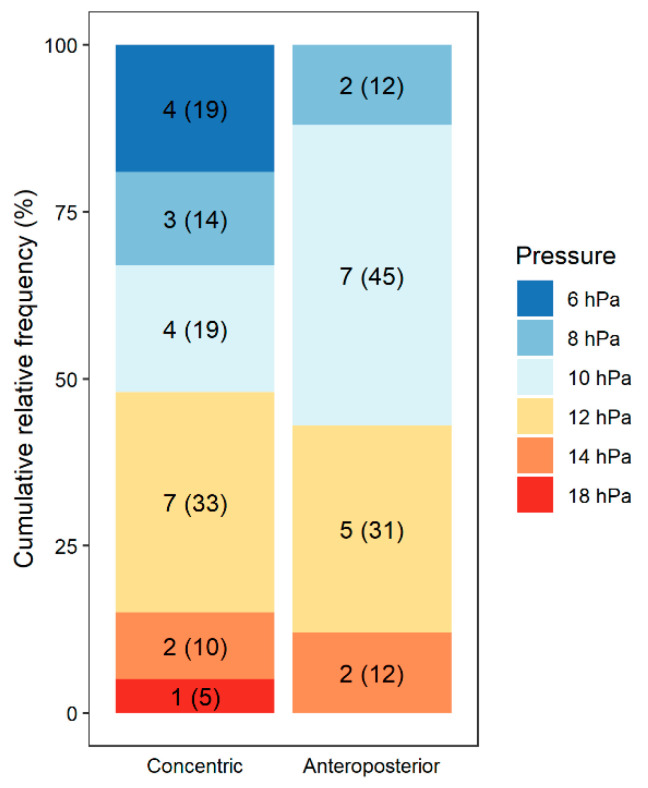
Analysis of the association between concentric and anteroposterior obstruction of the soft palate and the pressure at which improvement occurred.

Within the area of the soft palate, we observed all three types of obstruction (Table 2). Among the 20 patients with anteroposterior obstruction, 16 (80%) exhibited a median opening pressure of 10 hPa, while PAP had no effect on the remaining 4 (20%). Among the 33 patients with concentric obstruction, 21 (64%) exhibited a median opening pressure of 10 hPa, while PAP was ineffective in the remaining 12 (36%) (*p* = 0.343) (Figure 3, Table 3). In two patients with concentric obstruction, the application of higher pressure (10.0 hPa) resulted in a change of the obstruction to the anteroposterior configuration. Moreover, at a pressure of 12.0 hPa, these patients no longer had obstruction in this area.

**Figure 4:**
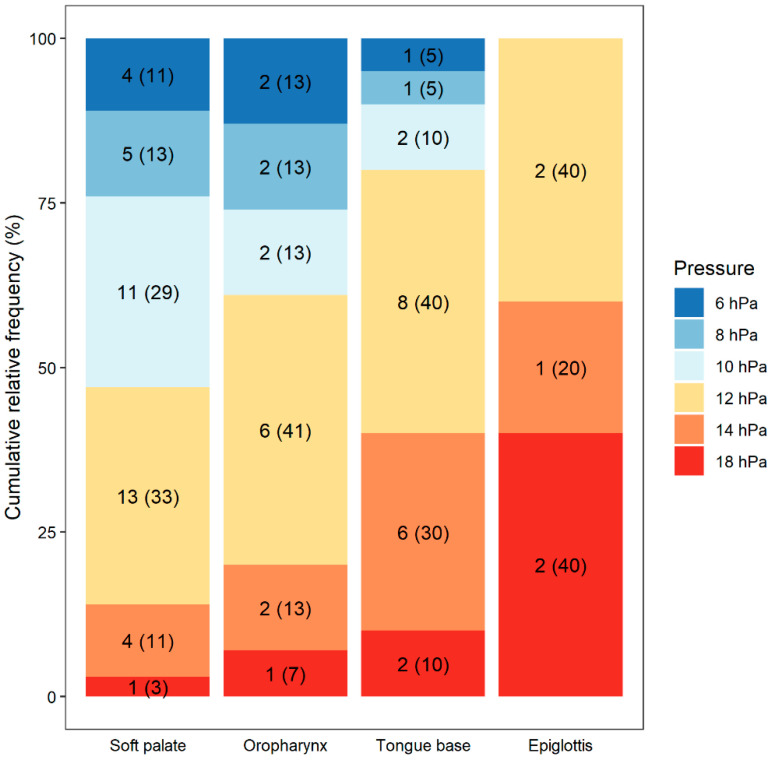
Analysis of the association between the level of the obstruction and the pressure at which improvement occurred.

PAP yielded improvement in all 15 patients (100%) with complete oropharyngeal obstruction; the median opening pressure was 12 hPa. Among the 23 patients with complete obstruction in the region of the base of the tongue, PAP was effective in 20 patients, and the median opening pressure was 12 hPa; in the remaining 3 patients, PAP had no effect at all, even with the highest tested pressure of 18.0 hPa. Among the 16 patients with epiglottic collapse, simultaneous PAP yielded improvement in 5 (31%), with a median opening pressure of 14 hPa. In the remaining 11 patients, PAP was ineffective, and resulted in the epiglottis being pushed even more strongly against the back wall of the pharynx, which was clinically correlated with persistence of apnea and simultaneous drop in SpO_2_. Figure 4 and Table 3 summarize the results regarding the mean opening pressure required to overcome obstructions in the individual upper airway regions.

### 3.3. Ineffectiveness of PAP

PAP was ineffective in 45% cases of complete soft palate obstruction, in 13% cases of complete tongue base obstruction, and in 69% cases of complete epiglottic collapse.

## 4. Discussion

DISE was first described in 1991 [13,14]. Since then, it has gradually become the most widely used method for upper airway examination while sleeping [8,15]. DISE is currently the main diagnostic method for determining the location of upper airways obstruction, and these findings can help with treatment optimization [6,7,8,16,17,18,19]. DISE is based on the assumption that the upper airway configuration differs when a person is awake versus sleeping, which has been proven by many authors [7,8,13,15]. DISE is mainly indicated in patients under consideration for surgical treatment of OSA [7,8]; it is also performed in patients who have either failed or are noncompliant with PAP therapy [20]. Over recent years, it has been demonstrated that DISE can be effectively used simultaneously with a PAP device, where optical control enables the direct monitoring of the effect of PAP [4,5]. However, only a limited number of studies have examined the importance of DISE in the current use of PAP [5,10,11].

PAP, which is considered a treatment of choice for patients suffering from moderate to severe OSA, is not easily accepted. Many diverse factors may cumulate leading to patients’ failure to initiate PAP or non-compliance [21]. There are numerous factors that may affect adherence to PAP treatment. For instance, several demographic and clinical variables, e.g., age, sex, BMI (body mass index), race, AHI, ESS score, and the presence of comorbidities, were shown to have an effect on compliance [22]. However, there are few studies that deal with the effect of PAP on individual locations of the upper airways. Too high an excess pressure, which in some cases is necessary to overcome an obstruction in a certain location of the upper airways, can be a particularly significant factor affecting PAP compliance [4,5].

According to current knowledge, overpressure ventilation is more effective in cases of obstructions of the soft palate and oropharynx, compared to obstructions of the base of the tongue or cases of epiglottic collapse [5,10,11]. In a study of 30 patients, Jung et al. confirmed that the oropharynx has a major influence on the effectiveness of PAP [10]. Accordingly, Schwab et al. assessed PAP effectiveness by conducting MRI examinations at different pressure levels (0.0, 5.0, 10.0, and 15.0 hPa), and observed that PAP had the greatest effect on lateral oropharyngeal walls [23].

Torre et al. also reported that PAP treatment is most effective for laterolateral oropharyngeal obstruction [5]. They found that in cases with complete concentric obstruction of the soft palate, the application of higher pressure led to a change in the anteroposterior configuration. With this type of obstruction, channels are laterally created, which enables airflow, such that PAP is effective. Their study also revealed that a mean pressure of 10.0 hPa was required for PAP to be effective in two-level obstruction of the soft palate and oropharynx. However, their work also describes the case of a patient with multilevel obstruction with hypertrophic palatine tonsils that completely blocked the upper airways. In that case, a pressure of up to 15.0 hPa was needed to open the upper airways. As this pressure was uncomfortable for the patient, a bilateral tonsillectomy was performed, which enabled the patient to tolerate the PAP pressure [5]. This case further illustrates the importance of PAP examination under the control of DISE. Direct visualization of the upper airways, with the simultaneous use of PAP, enables the detection of any anatomical abnormalities that could be surgically or orthodontically resolved to enable the reduction of PAP pressure with subsequent better tolerance of treatment. Torre et al. also reported that obstructions of the base of the tongue and epiglottic collapse require higher pressure to open the upper airways, with an average pressure of 15.0 hPa required to eliminate obstruction of the base of the tongue. PAP did not resolve primary epiglottic collapse, and was particularly ineffective in cases of laterolateral epiglottic obstruction, where the problem persists even with pressures higher than 15.0 hPa [5].

Lai et al. also reported that higher pressure is needed in cases where the obstruction is in the area of the base of the tongue, and that the area of the base of the tongue is essential for correct PAP setting [24]. In cases of primary epiglottic collapse, several studies have confirmed that increased PAP pressure leads to exacerbation of the obstruction, with the epiglottis being pushed onto the back wall of the pharynx [9,25,26,27]. Epiglottic collapse has been found in 15.0–31.4% of adult patients with OSA in whom PAP treatment was ineffective [2,26,28,29]. Unfortunately, epiglottic collapse is not observed during the examination of an awake patient. Our present results confirmed that PAP was most effective in the oropharynx, particularly in cases of its laterolateral obstruction, while PAP was least effective against obstructions caused by the epiglottis and the base of the tongue.

Overpressure must be correctly set to achieve a therapeutic effect, sufficient adherence to treatment, and a low level of side effects. Insufficient pressure reduces the number of apneas, but leaves hypopnea and awakening reactions, with the associated high cardiovascular risk. Insufficient positive pressure also fails to eliminate the subjective symptoms of OSA, resulting in low willingness to regularly use PAP. In contrast, excessively high overpressure can burden the patient with treatment-induced central sleep apnea, middle ear ventilation disorders, and other problems that ultimately contribute to inadequate treatment or low compliance [30].

Although PAP is considered the gold standard of OSA treatment, some patients experience no improvement and a persisting high residual AHI. Subjective problems can lead to a patient refusing to use overpressure and treatment failure. DISE with PAP is a very simple method that can be used to predict the success of PAP in individual patients. It can also reveal the anatomical and dynamic causes of PAP dysfunction, which may be resolved surgically to improve subsequent adherence to overpressure treatment [30].

This especially applies to cases involving the obstruction in the area of the epiglottis. Based on both our present results and previously published studies, we are inclined to recommend that when OSA is caused by epiglottis collapse, surgical treatment should be indicated. Possible surgical options include epiglottidectomy (total, partial, or V-shaped) or the more elegant and gentle method of transoral epiglottopexy [28,29,31,32,33,34]. Obstruction on the level of the base of the tongue also seems to be less suitable for overpressure treatment; therefore, additional care should be taken in setting up and following up treatment of these patients.

Our present results are limited by the small number of patients, the effect of the presence of optical fibers on PAP, and by the use of the VOTE classification, which is a subjective evaluation system. However, it should be noted that this classification is recognized worldwide, and we tried to account for its limitations by having two experienced physicians independently evaluate our findings. Subjective video closure analysis could be objectified by implementing DISE with type 3 PSG monitoring [35]. Another limitation of the study is that the patient position was only supine and only an oronasal mask was used. Some studies have suggested that higher PAP pressures may be required when this type of mask is used [36]. For technical reasons, it was only possible to use an oronasal mask. Only this type of mask is able to connect to a “connecting elbow” with a perforation for the entry of the endoscope. The short period of DISE and the lack of different sleep phases (REM, NREM) were other limitations, but the depth of anesthesia was monitored using the BIS (bispectral index) within the specified range.

## 5. Conclusions

PAP performed in combination with DISE is a simple, valid, safe, effective, and easy-to-use diagnostic method, which directly allows visualization of the effects of PAP ventilation on different types of upper airway obstructions at specific sites. It can be helpful for identifying anatomic and dynamic reasons for PAP intolerance or ineffectiveness. Our present results indicated that PAP was most effective in cases of laterolateral oropharyngeal obstruction, while PAP was least effective in cases of obstruction on the level of the epiglottis and the base of the tongue. DISE and PAP could be used to improve PAP treatment compliance and effectiveness.

## Figures and Tables

**Table 1:** Demographic data, entrance limited polygraphy, and ENT examination while conscious.

	Median (IQR) or n (%)
Age, years	46.0 (39; 55)
BMI, kg/m^2^	29.2 (27.4; 31.3)
AHI	26.4 (18.9; 31.8)
T90, %	2.3 (0.6; 9.1)
Soft palate obstruction	53 (94.6)
Oropharynx obstruction	13 (23.2)
Tongue base obstruction	39 (69.6)
Epiglottic pathology	---
Mallampati	
I	2 (3.6)
II	10 (17.9)
III	30 (53.6)
IV	14 (25.0)
Friedman	
0	4 (7.1)
1	28 (50.0)
2	22 (39.3)
3	2 (3.6)

The values represent the median and interquartile range (IQR) or absolute and relative frequencies (%). AHI = apnea–hypopnea index; T90 = time under SaO_2_ < 90%.

**Table 2:** Degree and type of obstruction and analysis of the overall improvement with PAP.

	Soft Palate	Oropharynx	Tongue Base	Epiglottis	*p*
Obstruction—degree					
Complete	51 (91.1)	15 (26.8)	23 (41.1)	16 (28.6)	
Partial	4 (7.1)	27 (48.2)	17 (30.4)	5 (8.9)	
No	1 (1.8)	14 (25.0)	16 (28.6)	35 (62.5)	
Obstruction—type					
Concentric	33 (58.9)	---	---	---	
Laterolateral	2 (3.6)	42 (75.0)	---	2 (3.6)	
Anteroposterior	20 (35.7)	---	40 (71.4)	19 (33.9)	
No	1 (1.8)	14 (25.0)	16 (28.6)	35 (62.5)	
Improvement	38/51 (74.5)	15/15 (100.0)	20/23 (87.0)	5/16 (31.3)	<0.001

The numbers represent absolute frequencies and relative frequencies (%). The *p* value was obtained using the chi-square test for equality of proportions.

**Table 3:** Median opening pressure in individual areas, and analysis of the inefficiency of the PAP.

Level of Obstruction	Type of Obstruction	Median Opening Pressure (hPa)	Ineffectiveness of PAP (% Patients)
Soft palate	Anteroposterior	10	20.0
	Concentric	10	36.4
Oropharynx	Laterolateral	12	0.0
Tongue base	Anteroposterior	12	13.0
Epiglottis	Anteroposterior (collapse)	14	68.8

## Data Availability

All available data presented.
